# ID1对非小细胞肺癌EGFR-TKI耐药的影响

**DOI:** 10.3779/j.issn.1009-3419.2016.12.10

**Published:** 2016-12-20

**Authors:** 昱辰 鲍, 印敏 赵, 斌 陈, 洁 罗, 沁芳 邓, 辉 孙, 博雄 谢, 崧雯 周

**Affiliations:** 1 200433 上海，同济大学附属上海市肺科医院肿瘤科 Department of Oncology, Pulmonary Disease Hospital of Tongji University, Shanghai 200433, China; 2 200433 上海，同济大学附属上海市肺科医院胸外科 Department of Thoracic Surgery, Pulmonary Disease Hospital of Tongji University, Shanghai 200433, China

**Keywords:** 肺肿瘤, 分化抑制因子, EGFR-TKI, 获得性耐药, Lung neoplasms, ID1, EGFR-TKI, Drug resistance

## Abstract

**背景与目的:**

非小细胞肺癌(non-small cell lung cancer, NSCLC)是当今世界上发病率和死亡率最高的恶性肿瘤之一，而表皮生长因子受体-酪氨酸激酶抑制剂(epidermal growth factor receptor-tyrosine kinase inhibitors, EGFR-TKI)整体有效率为30%-40%，无进展生存期(progression-free survival, PFS)为12个月。但EGFR-TKI在临床中的耐药现象也很普遍，严重影响了其抑瘤作用。因此，克服耐药、寻找一种新的与肺癌耐药相关的预后因子势在必行。本研究旨在通过体内外实验探讨DNA结合抑制因子1(differentiation inhibitory factor 1, ID1)与NSCLCEGFR-TKI耐药之间的关系，看其是否有统计学意义，并初步探讨其机制。

**方法:**

免疫组化(immunohistochemistry, IHC)检测手术标本(肺癌组织和癌旁组织)1D1的表达；qRT-PCR、Western-blot检测并比较肺癌细胞敏感株与耐药株中ID1的表达变化；MTT检测吉非替尼对ID1慢病毒载体处理肺癌细胞的细胞增殖情况，将肺癌细胞接种至裸鼠腋下，待肿瘤生长至一定体积使用吉非替尼治疗，估算肿瘤体积。

**结果:**

ID1在肺癌组织中的表达明显高于正常组织(*P* < 0.05)；ID1的表达与NSCLC EGFR-TKI耐药呈正相关(*P* < 0.05)。

**结论:**

ID1在NSCLC中高表达，并且参与了NSCLC EGFR-TKI的耐药，其机制可能与STAT3磷酸化程度增加有关。

## Abstract (en)

**Background and objective:**

Non-small cell lung cancer (NSCLC) presents the highest morbidity and mortality among malignant tumors worldwide. The overall effective rate of epidermal growth factor receptor-tyrosine kinase inhibitor (EGFR-TKI) is 30% to 40%, and PFS (progression-free sruvival) is 12 months. However, EGFR-TKI resistance is typical in clinical observations, and this phenomenon significantly affects tumor suppression. To overcome this resistance, a new prognostic factor associated with lung cancer drug resistance should be discovered. This study investigated the relationship between the inhibitor of differentiation 1 (ID1) and non-small cell lung cancer EGFR-TKI resistance in vivo and in vitro to determine any statistical significance and discuss the underlying mechanism.

**Methods:**

Western blot and qRT-PCR were used to quantify the expression of ID1 in lung cancer. IHC was used to detect the expression of ID1 in pathological tissues (lung cancer tissues and adjacent tissues). MTT was used to detect cell proliferation, in which the cells were treated with gefitinib after being transfected by ID1 slow virus vector. Lung cancer cells were inoculated in nude mice until the tumor diameter grew to certain measurement. Gefitinib treatment was started, and the tumor volume was estimated.

**Results:**

ID1 was highly expressed in NSCLC (*P* < 0.05). Both ID1 expression and drug resistance of EGFR-TKI in NSCLC were positively correlated (*P* < 0.05). The treatment group with gefitinib showed obviously less expression than the control group.

**Conclusions:**

ID1 is highly expressed in NSCLC. ID1 expression was positively related to drug resistance of EGFR-TKI in NSCLC. Gefitinib can be used to effectively treat NSCLC, and the mechanism may be associated with an increased level of STAT3 phosphorylation.

肺癌的发病率和死亡率很高，严重威胁人类健康^[1, 2]^。在我国城市中肺癌死亡率已上升为第一位^[3]^。随着分子生物学的研究进展，靶向药已成为治疗非小细胞肺癌的重要手段^[4]^。然而，即使对于敏感人群，第一代表皮生长因子受体-酪氨酸激酶抑制剂(epidermal growth factor receptor-tyrosine kinase inhibitors, EGFR-TKI)(包括易瑞沙、特罗凯、凯美纳)或早或晚仍会出现获得性耐药，导致疾病进展^[5, 6]^，因此，如何克服耐药提高疗效已成为靶向药治疗的重要瓶颈^[7, 8]^。分化抑制因子(differentiation inhibitory factor, ID)是一种负性调节因子^[9, 10]^。近年的研究表明，ID蛋白在肿瘤发生及发展过程中发挥了重要作用^[11]^。ID1作为ID蛋白家族成员，在多种肿瘤中呈高表达，是一种潜在的癌基因^[12, 13]^。有研究^[14, 15]^认为ID1与肺癌靶向药耐药相关，但相关报道甚少，具体分子机制未明。

## 1 材料与方法

### 1.1 主要试剂与仪器

肺腺癌细胞株H522、H1975、A549、PC-9、PC-9R由上海市肺科医院中心试验室提供。其中PC-9为EGFR-TKI敏感株，PC-9R为EGFR-TKI获得性耐药株，H1975为T790M突变EGFR-TKI耐药株，A549、H522为EGFR-TKI原发耐药株，获取我院肺癌手术患者癌组织及癌旁组织。青霉素、链霉素购自上海先锋药业公司；Trizol试剂购自Invitrogen公司；SYBR Green PCR试剂盒购自TAKARA公司；RNaseI购自Fermentas公司；兔抗人单克隆抗体购自Abcam公司；BSA购自北京索莱宝生物科技有限公司；吉非替尼购自大连美仑；Real-time检测仪购自ABI公司；流式细胞仪购自Backman Coulter公司。

### 1.2 细胞培养

H522、H1975、A549、PC-9、PC-9R细胞用10%DMEM含双抗培养液在细胞恒温培养箱(温度为37 ℃且含5%CO_2_)中培养，次日换液。培养基为90%EMEM，10%胎牛血清。0.25%胰酶-EDTA消化传代，所有试验均采用对数生长期细胞。

### 1.3 ID1 siRNA慢病毒载体及ID1过表达慢病毒载体的构建

ID1 siRNA慢病毒载体构建：shRNA序列：5’-CATGAACGGCTGTTACTCA-3’；病毒滴度：8×10^8^ U/mL，同时构建阴性对照慢病毒，ID1过表达慢病毒载体的构建：ID1基因片段引物：上游5’-GAGGATCCCCGGGTACCGGTCGCCACCATGAAAGTCGCCAGTGGCAG-3’；下游5’-TCCTTGTAGTCCATACCGCGACACAAGATGCGATC-3’。病毒滴度：2×10^8^ U/mL，同时构建阴性对照慢病毒。胰酶消化各组肺腺癌细胞，制成细胞悬液；显微镜下数出细胞总数，荧光显微镜观察荧光表达情况。

### 1.4 MTT法检测细胞增殖

细胞计数，调整细胞浓度，每孔悬液约100 μL，以5, 000个/孔接种到96孔细胞培养板中，37 ℃、CO_2_培养24 h；弃培养基，加入含不同浓度吉非替尼(0 μmol/L、0.01 μmol/L、0.05 μmol/L、0.1 μmol/L、0.5 μmol/L、1 μmol/L、5 μmol/L、10 μmol/L、20 μmol/L、30 μmol/L、40 μmol/L、50 μmol/L)的培养基继续培养72 h；每孔加入5 mg/mL MTT溶液20 μL，37 ℃培养4 h；吸去上清液，加入200 μL二甲基亚砜(DMSO)，使结晶物充分溶解；采用酶标仪检测570 nm的吸光度(*A*)值，每组设4个复孔；按以下公式计算细胞增殖率：[(空白组吸光度平均值-DMSO空白组平均值)-(各组平均值-DMSO空白组平均值)]/(空白组平均值-DMSO空白组平均值) ×100%=抑制率；采用直线回归方法计算药物的半抑制浓度(half maximal inhibitory concentration, IC_50_)值，实验重复3次。

### 1.5 Real-Time PCR检测各组肿瘤细胞ID1 mRNA的表达情况

去对数生长的各组肺腺癌细胞，Trizol法提取RNA，逆转录制备cDNA。将制备好的cDNA进行PCR扩增，ID1上游引物序列：5’-AAACGTGCTGCTCTACGACA-3’，ID1下游引物序列：5’-GGAACGCATGCCGCCT-3’，GAPDH上游引物序列：5’-CACCCACTCCTCCACCTTTG-3’，GAPDH下游引物序列：5’-CCACCACCCTGTTGCTGTAG-3’；95 ℃变性10 min后，按下述条件扩增40个循环，95 ℃，15 s；60 ℃，45 s；60 ℃延伸1 min。

### 1.6 免疫组化检测ID1蛋白的表达

选取标本经10%甲醛固定后，常规石蜡包埋、切片，厚度4 μm。采用免疫组化SP法，用PBS液代替一抗作为阴性对照，按照试剂说明书进行操作。结果判断：所有切片均采用双盲法由两位病理科医师独立阅片。ID1阳性表达均定位于细胞浆和细胞膜，呈浅黄色、黄色或棕黄色。随机选择10个高倍镜视野(400倍)，每个视野连续计数100个细胞，共计数1, 000个细胞。最后表达以染色强度和阳性细胞率的得分之和进行判断：无染色记0分，弱染色记1分，中等染色记2分，强染色记3分；阳性细胞率 < 5%记0分，5%-25%记1分，26%-50%记2分， > 50%记3分。上述两项评分相加， < 3分为阴性，≥3分为阳性。

### 1.7 Western blot检测相关蛋白的表达

取对数生长期的经siRNA慢病毒载体及ID1过表达慢病毒载体转染的H522、H1975、A549、PC-9、PC-9R细胞，收集细胞裂解提取蛋白。BCA法测定细胞裂解物的蛋白含量，取等量蛋白质以12% SDS-PAGE法分离并转移至PVDF膜上，以单克隆抗体4 ℃过夜孵育以检测目标蛋白(p-ERK、ERK、p-AKT、AKT、p-STAT3、STAT3、p-EGFR、EGFR)，GAPDH作为内参。洗去一抗，以HRP连接的二抗于室温孵育2 h，洗涤后以ECL试剂盒显示免疫印迹条带。

### 1.8 动物实验

将肺腺癌细胞PC-9、PC-9/ID1-OE、PC-9/R、PC-9/R/ID1-KD细胞接种至75 cm2细胞培养瓶中，待细胞扩增至90%，胰酶消化，接种至BALB/c-nu裸鼠腋下；每只接种1×10^8^个细胞。每株肺腺癌实验动物各设空白对照和吉非替尼两组。待肿瘤直径生长至3 mm左右时，空白对照组使用生理盐水灌胃治疗，吉非替尼组使用吉非替尼灌胃治疗(剂量为2 mg/kg/d)，每天灌胃一次，持续4周；4周后，颈椎脱臼法处死，剥离肿瘤组织，测量肿瘤大小。

### 1.9 统计学方法

采用SPSS 20.0统计学软件进行统计分析，所有数据均以Meab±SD表示，单变量两组间资料比较采用*t*检验，以*P* < 0.05为差异有统计学意义。

## 2 结果

### 2.1 ID1在肺癌细胞株中高表达

肺癌细胞株H522、H1975、A549、PC-9、PC-9R中的ID1 mRNA均有表达(图 1)。肺癌组织ID1 mRNA的表达比癌旁组织中高，有统计学差异(*P* < 0.05)(图 2A)。肺癌组织ID1蛋白的表达比癌旁组织中表达高，有统计学差异(*P* < 0.05)(图 2B)。

**1:**
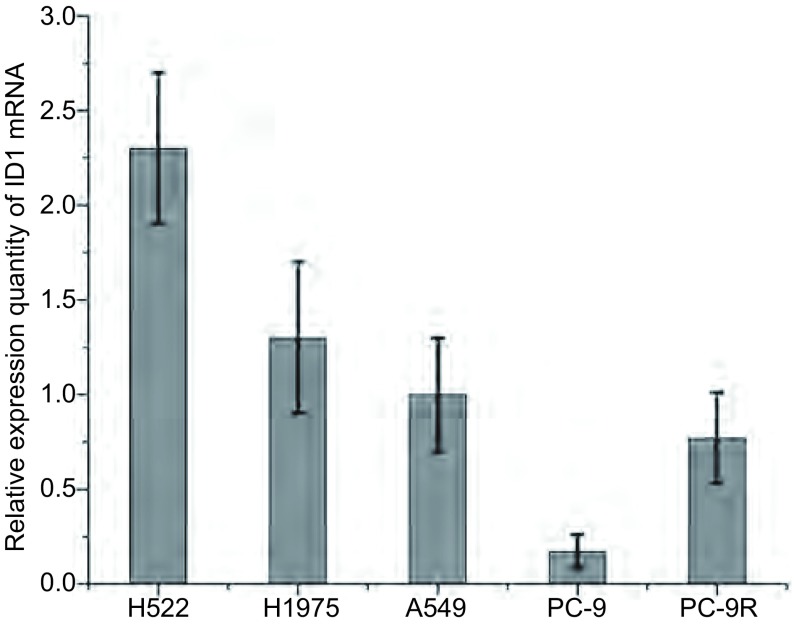
肺癌细胞株H522、H1975、A549、PC-9、PC-9R中的ID1 mRNA相对表达量(*P* < 0.05) Relative expression of ID1 mRNA in H522, H1975, A549, PC-9 and PC-9R lung cancer cell lines (*P* < 0.05)

**2:**
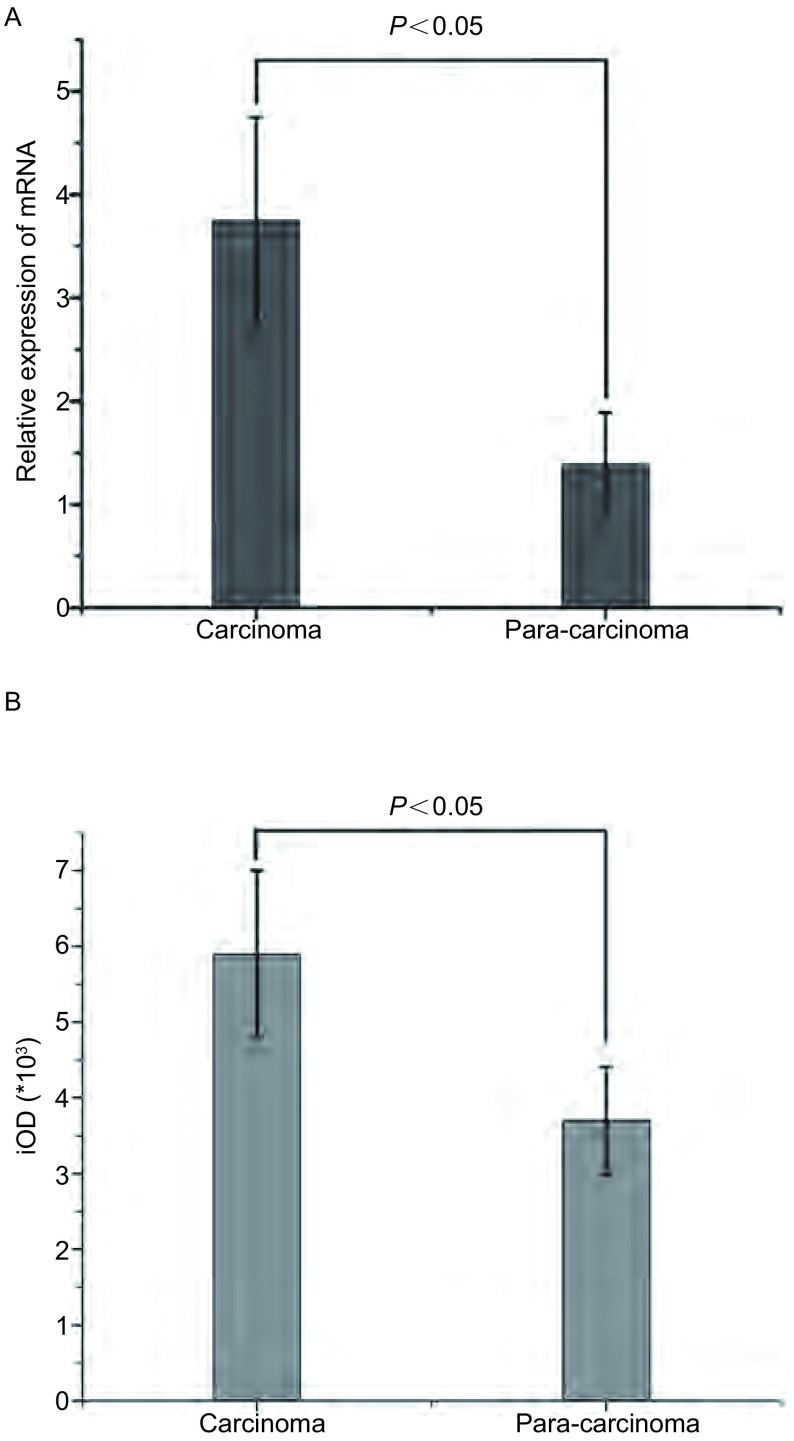
ID1在肺癌组织与癌旁组织的相对表达量(*P* < 0.05)。A：肺癌组织ID1 mRNA的表达量比癌旁组织中ID1 mRNA的表达量高(*P* < 0.05)；B：肺癌组织ID1蛋白的表达比癌旁组织中ID1蛋白的表达高(*P* < 0.05)。 Relative expression of ID1 in lung cancer tissues and adjacent tissues. A: The expression of ID1 mRNA in lung cancer tissues is relatively higher than that in adjacent tissues (*P* < 0.05); B, C: The expression of ID1 protein in lung cancer tissues is higher than that in adjacent tissues (*P* < 0.05). iOD: integral optical density.

### 2.2 ID1与肺癌耐靶向药的关系

PC-9R细胞中ID1 mRNA表达量高于PC-9细胞，且具有统计学意义(*P* < 0.05)(图 1，图 3)。根据荧光定量PCR的实验结果，选择PC-9细胞株感染ID1过表达慢病毒载体(ID1-OE)，H522、H1975、A549、PC-9R细胞株感染ID1干扰慢病毒载体。72 h后用荧光显微镜观察，各组病毒感染率均大于90%，符合后续实验要求。MTT法检测吉非替尼对各组细胞增殖抑制作用。实验结果显示PC-9细胞感染ID1过表达慢病毒后，对吉非替尼的IC_50_值为0.64 μmol/L，较对照组(0.05 μmol/L)有一定程度的上升。PC-9R细胞感染ID1干扰慢病毒后，对吉非替尼的IC_50_值为1.14 μmol/L，较对照组(5.05 μmol/L)有一定程度的下降(表 1)。而A549、H522、H1975细胞在感染ID1干扰慢病毒后，对吉非替尼的IC_50_值无明显改变。

**3:**
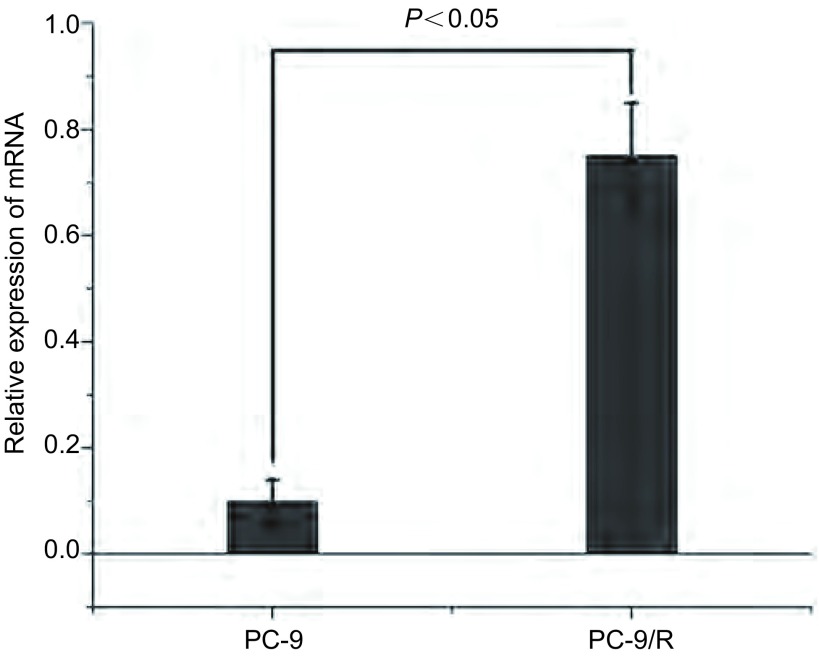
PC-9R细胞中ID1 mRNA表达量高于PC-9细胞(*P* < 0.05)。 The expression of ID1 mRNA in PC-9/R cell lines is higher than that in PC-9 cell lines (*P* < 0.05).

**1:** 组肿瘤细胞IC_50_值和吉非替尼处理后凋亡率 IC_50_ value in groups of tumor cells and apoptosis rate after treatment with gefitinib

Group	IC_50_	Apoptosis rate after treatment with gefitinib of 10 μmol/L
PC-9 blank control group	0.05	65
PC-9 NC group	0.05	65
PC-9 ID1 OE	0.64	55
PC-9/R blank control group	5.84	49
PC-9/R NC group	5.05	52
PC-9/R-ID1-siRNA	1.14	58
*P*	< 0.05	< 0.05
PC-9 NC: PC-9 negative control; PC-9 ID1 OE: PC-9 ID1 over expression; PC-9R NC: PC-9R negative control. IC_50_: half maximal inhibitory concentration.				

### 2.3 各组细胞株在感染ID1干扰慢病毒后，ID1的表达情况

慢病毒感染后，H522、H1975、PC-9R、A549细胞中ID1表达下降，PC-9细胞中ID1表达上升(图 4)。ID1-siRNA慢病毒感染PC-9R后，p-ERK、p-AKT、p-STAT3、p-EGFR有不同程度下降，ERK、AKT、STAT3、EGFR无明显改变；吉非替尼处理后，ID1-siRNA组ERK、AKT、EGFR磷酸化程度较对照组明显降低。ID1-OE慢病毒感染PC-9后，磷酸及非磷酸ERK、AKT、STAT3、EGFR均无明显改变；吉非替尼处理后，PC-9-ID1-OE组AKT、STAT3磷酸化程度较对照组有一定程度升高(图 5)。

**4:**
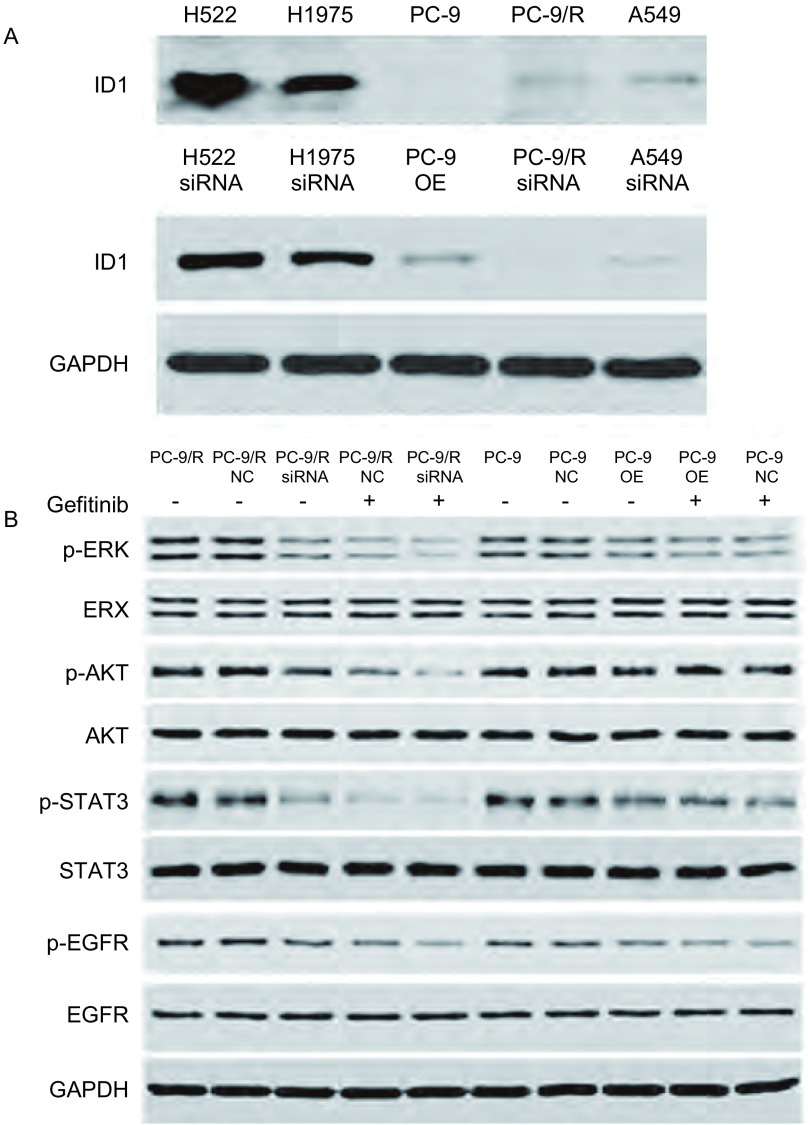
慢病毒感染后，各组细胞ID1的表达情况以及PC-9R中不同信号通路的酶表达情况。A：慢病毒感染后，H522、H1975、PC-9R、A549细胞中ID1表达下降，PC-9细胞中ID1表达上升；B：ID1-siRNA慢病毒感染PC-9R后，p-ERK、p-AKT、p-STAT3、p-EGFR有不同程度下降，ERK、AKT、STAT3、EGFR无明显改变；吉非替尼处理后，ID1-siRNA组ERK、AKT、EGFR磷酸化程度较对照组明显降低。ID1-OE慢病毒感染PC-9后，磷酸及非磷酸ERK、AKT、STAT3、EGFR均无明显改变；吉非替尼处理后，PC-9-ID1-OE组AKT、STAT3磷酸化程度较对照组有一定程度升高。 After slow viral infection, groups of cells expressed the ID1 enzyme in different signaling pathways of PC-9R. A: After infection of ID1-siRNA; ID1 expression decreases in H522, H1975, PC-9R, and A549 while increasing in PC-9; B: After infection of ID1-siRNA in PC-9R, p-ERK, p-AKT, p-STAT3 and p-EGFR decreased, whereas ERK, AKT, STAT3 and EGFR showed no change. Following gefitinib treatment, the extent of phosphorylation of ERK, AKT and EGFR in ID1-siRNA is lower than that in the control group. After infection of ID1-OE in PC-9, the extent of phosphorylation or non-phosphorylation of ERK, AKT and EGFR showed no change. After gefitinib treatment, the extent of phosphorylation of AKT and STAT3 in PC-9-ID1-OE is higher than that in the control group.

**5:**
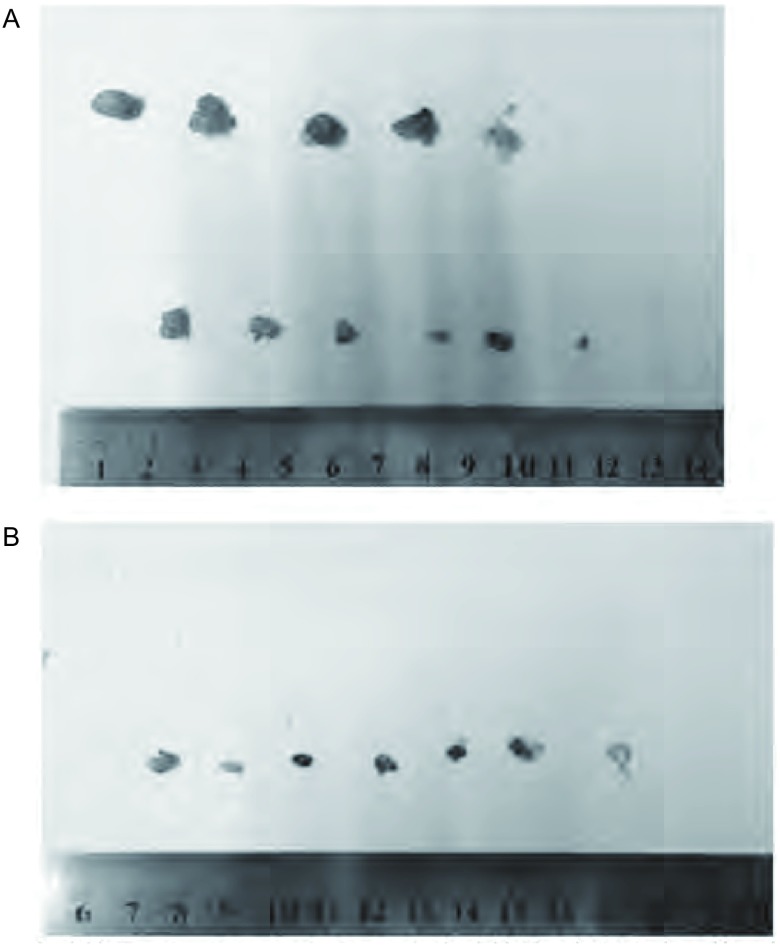
动物实验结果。A：PC-9/R(ID1-OE)实验结果(标尺上方，第一行：对照组剥离肿瘤；第二行：灌胃组剥离肿瘤)；B：PC-9R(ID1-siRNA)实验结果(标尺上方，第一行：对照组剥离肿瘤，第二行：灌胃组因肿瘤消失，故空行)，值得注意的是，ID1基因沉默后，PC-9R(ID1 siRNA)肿瘤生长极为缓慢，28天仅增长0.9倍。 Animal experiment. A: Experimental result of PC-9/R (ID1-OE) (Above scale, first line: control group stripping tumor, second line: lavage group stripping tumor); B: Experimental result of PC-9/R (ID1-siRNA) (Above scale, first line: control group stripping tumor, second line: when the tumor disappears, the lavage group is zero). Notably, with silence of the ID1 gene, tumor growth is very slow in PC-9R (ID1 siRNA), which grew only 0.9 times after 28 days.

### 2.4 动物实验结果

PC-9实验组肿瘤平均体积为50.8 mm^3^，对照组肿瘤平均体积3, 283.6 mm^3^，肿瘤抑制率为98.5%；PC-9(ID1 OE)实验组肿瘤平均体积为465.5 mm^3^，对照组肿瘤平均体积为1, 084.7 mm^3^，肿瘤抑制率为57.1%，组间存在统计学差异(表 2)。PC-9/R实验组肿瘤平均体积为81.2 mm^3^，PC-9/R对照组肿瘤平均体积为371.8 mm^3^(图 5A)，肿瘤抑制率为78.2%；PC-9/R(ID1 siRNA)实验组肿瘤平均体积为0，对照组肿瘤平均体积为85.3 mm^3^(图 5B)，肿瘤抑制率为100%，组间存在统计学差异(表 2)，值得注意的是，ID1基因沉默后，PC-9/R肿瘤生长极为缓慢，28 d仅增长0.9倍。

**2:** 吉非替尼治疗组与对照组的瘤体体积与抑瘤率 Gefitinib treatment group and control group in the volume of tumors and inhibitory rate

Group	Average tumor volume (mm^3^)		Inhibition rate
	Treatment group of gefitinib	Control group	
PC-9	50.8	3, 283.6	98.5%
PC-9 ID1 OE	465.5	1, 084.7	57.1%
PC-9/R	81.2	371.8	78.2%
PC-9/R ID1 siRNA	0.0	85.3	100%
*P*	< 0.05	< 0.05	< 0.05

## 3 讨论

本研究证实了ID1参与非小细胞肺癌对EGFR-TKI耐药，且与手术患者预后相关。在探讨ID1是否参与肺癌靶向药耐药的相关研究中，我们检测了PC9及PC-9/R、H1975、A549、H522多组肺癌细胞株中ID1 mRNA。发现PC-9细胞中ID1 mRNA相对表达量较低，而PC-9/R细胞则明显升高，说明ID1与肺癌EGFR-TKI耐药相关。同时，发现H1975、A549、H522多组肺癌细胞株中ID1高表达，故对非小细胞肺癌手术标本免疫组化及RT-PCR检测，提示ID1在肺癌组织中较正常组织明显升高(*P* < 0.05)；分层分析显示，病理类型对ID1表达有意义，在鳞癌患者中呈高表达；分期及性别与ID1表达无相关性；1年OS与ID1表达分析提示，ID1与肺癌预后相关。进一步探讨ID1与非小细胞肺癌对EGFR-TKI耐药的相关性：使用慢病毒干扰技术，过表达及沉默肺癌细胞株ID基因的表达，再检测ID1蛋白的表达变化。结果显示：PC-9细胞感染ID1过表达慢病毒后，对吉非替尼的IC_50_值为0.64 μmol/L，较对照组(0.05 μmol/L)有一定程度的上升，说明PC-9敏感性降低；PC-9/R细胞感染ID1干扰慢病毒后，对吉非替尼的IC_50_值为1.14 μmol/L，较对照组(5.05 μmol/L)有一定程度的下降，说明PC-9/R耐药性降低(表 1)。荷瘤裸鼠实验结果：PC-9 ID1-OE吉非替尼用药组肿瘤体积大于PC-9吉非替尼用药组。同时发现，吉非替尼处理后，ID1-siRNA组ERK、AKT、EGFR磷酸化程度较对照组明显降低，ID1-OE组AKT、STAT3磷酸化程度较对照组有一定程度升高。这说明：ID1表达量与肺癌EGFR-TKI耐药性呈正相关；STAT3可能通过磷酸化机制参与EGFR-TKI耐药。有研究^[16]^发现，STAT3的siRNA或抑制剂通过抑制STAT3激活，增强了肺癌细胞对吉非替尼的敏感性。但目前STAT3介导吉非替尼耐药的机制还不是很明确，需要进一步研究。

本研究通过体内外实验从细胞、动物到临床标本深入探讨了ID1与肺癌之间的关系，证实ID1在肺癌组织中高表达，其中以腺癌尤为明显，且ID1参与非小细胞肺癌对EGFR-TKI的获得性耐药。ID1或可成为非小细胞肺癌有价值的检测项目^[17]^；ID1参与肺癌对EGFR-TKI的耐药，可能与STAT3的磷酸化有关，具体机制还有待我们接下来更深入地研究^[17]^。
